# Photo-dependent cytosolic delivery of shRNA into a single blastomere in a mouse embryo

**DOI:** 10.1038/s41598-023-40361-9

**Published:** 2023-08-11

**Authors:** Yuka Ikawa, Takuya Wakai, Hiroaki Funahashi, Tet Htut Soe, Kazunori Watanabe, Takashi Ohtsuki

**Affiliations:** 1 Department of Interdisciplinary Science and Engineering in Health Systems, Okayama University, 3-1-1 Tsushimanaka, Okayama, 700-8530 Japan; 2 Department of Animal Science, Graduate of Environmental and Life Science, Okayama University, Okayama, 700-8530 Japan

**Keywords:** Biological techniques, Biotechnology

## Abstract

Single-cell-specific delivery of small RNAs, such as short hairpin RNA (shRNA) and small noncoding RNAs, allows us to elucidate the roles of specific upregulation of RNA expression and RNAi-mediated gene suppression in early embryo development. The photoinduced cytosolic dispersion of RNA (PCDR) method that we previously reported can introduce small RNAs into the cytosol of photoirradiated cells and enable RNA delivery into a single-cell in a spatiotemporally specific manner. However, the PCDR method has only been applied to planer cultured cells and not to embryos. This study demonstrated that the PCDR method can be utilized for photo-dependent cytosolic shRNA delivery into a single blastomere and for single blastomere-specific RNA interference in mouse embryos. Our results indicate that PCDR is a promising approach for studying the developmental process of early embryogenesis.

## Introduction

Fertilized eggs undergo repeated cleavages and pass through embryonic stages such as the 2-cell, 4-cell, 8-cell, morula, and blastocyst stages before implantation^1,2^. The developmental process up to the blastocyst stage is a critical period that determines implantation in the uterus^3^. In early development, spatiotemporal-specific RNA and protein expression is thought to contribute to asymmetric cell division and early cell differentiation^4^. Single-blastomere transcriptomic analyses have revealed that the molecular symmetry-breaking process already begins as early as the 2-cell stage^5^. However, much remains unknown about the molecular mechanisms of early embryonic development^4,6,7^. To investigate the role of the temporal and local upregulation of each RNA in early embryogenesis, single-cell-specific RNA delivery methods are useful. In addition, single-cell-specific delivery of small interfering RNA (siRNA) or short hairpin RNA (shRNA) allows us to elucidate the effects of RNA interference (RNAi)-mediated spatiotemporal gene knockdown on early development. Microinjection is capable of single-cell-specific RNA injection, but mechanical damage to the cell is inevitable^8^. Another methodology that may allow for embryonic single-cell-specific RNA delivery is photo-dependent RNA internalization^9,10^.

Previously, we developed an RNA delivery method, photoinduced cytosolic dispersion of RNA (PCDR), using photosensitive RNA carriers^11–13^. The RNA carrier, TatU1A-Alexa546, is composed of HIV TAT-derived cell-penetrating peptide^14^, the RNA-binding domain of U1 small nuclear ribonucleoprotein A (U1A)^15^, and Alexa Fluor 546 (Alexa546) as a photosensitizer. The U1A RNA-binding domain binds to a specific 9-nt RNA sequence. Thus, TatU1A-Alexa546 can deliver RNAs containing the U1A binding sequence. The PCDR procedure is as follows: TatU1A-Alexa546 delivers an RNA to mammalian cells by endocytosis, but the RNA is trapped by endosomes and does not function. Photoirradiation of the photosensitizer generates singlet oxygen, which induces endosomal escape and cytosolic dispersion of the RNA in the irradiated cell^16^. Thus, via the PCDR method, small RNAs having a U1A binding sequence, such as siRNA, shRNA, and precursor miRNA can be photo-dependently delivered into the cytosol in a time- and region-specific manner^17–19^. However, the PCDR method has only been applied to two-dimensionally cultured cells, not to three-dimensional cell assemblies such as embryos. Thus, here we attempted to apply the PCDR method to single-cell-specific cytosolic RNA delivery and the photoinduction of RNAi in mouse embryos.

## Materials and methods

### Embryo collection and culture

Mouse zygotes were collected from superovulated 8- to 12-week-old CD1 female mice. Females were injected with 7.5 IU of pregnant mare serum gonadotropin, followed by 7.5 IU of human chorionic gonadotropin 48 h after pregnant mare serum gonadotropin. Embryos were obtained by mating the superovulated females with CD1 males. For isolation of fertilized zygotes, superovulated females were euthanized by cervical dislocation 20 h post-human chorionic gonadotropin treatment, and zygotes were dissected out of the ampulla in the oviduct. The embryo-cumulus complexes were treated with 300 µg/mL of hyaluronidase to disperse the cumulus cells, washed in M2 medium, and cultured at 37 °C in 5% CO_2_. All experimental protocols were approved by the Animal Care and Use Committee of Okayama University (permission number; OKU-2021234). All methods were carried out in accordance with relevant guidelines and regulations of Okayama University. All methods are reported in accordance with ARRIVE guidelines (https://arriveguidelines.org) for the reporting of animal experiments.

### Preparation of TatU1A-Alexa546

RNA carrier proteins with C-terminal Cys residues (TatU1A-C) were purified as described previously^11^. The purified TatU1A-C protein (20–30 µM) and Alexa Fluor 546 C_5_ maleimide (25 µM; Invitrogen, Waltham, MA, USA) were mixed in T buffer (20 mM HEPES–KOH (pH 7.6), 115 mM NaCl, 5.4 mM KCl, 1.8 mM CaCl_2_, 0.8 mM MgCl_2_, and 13.8 mM glucose) and incubated at 25 °C for 1 h. The reactant (TatU1A-Alexa546) was purified on a NAP-10 column (Cytiva, Japan). The protein concentration of TatU1A-Alexa546 was determined by protein assay CBB solution (Nacalai tesque, Japan).

### shRNAs

Anti-EGFP shRNA (shGFP; 5′-GGC UAC GUC CAG GAG CGC ACA UUG CAC UCC GUC GCG UCC UGG ACG UAG CCU U-3′) and scrambled shRNA (shCtrl; 5′-GAG CGA CUA AAC ACA UCA ACA UUG CAC UCC GUU GAU GUG UUU AGU CGC UCU U-3′) were purchased from JBioS, Japan (the U1A binding sequence is underlined). Fluorescein amidite (FAM)-labeled shRNA (5′-GAU UAU GUC CGG UUA UGU ACA UUG CAC UCC GU CAU AAC CGG ACA UAA UCdT dT-FAM-3′), which is anti-luciferase but nonspecific to mammalian genes, was purchased from Hokkaido System Science, Japan. Before use, all shRNAs were heated at 85 °C for 1 min, followed by annealing by − 1 °C/s to 4 °C.

### Intra-blastomere RNA delivery in zona-free embryos by PCDR method

TatU1A-Alexa546 (2 μM), shRNA (200 nM shCtrl for TatU1A-Alexa546-localization analyses, and 300 nM shGFP for knockdown experiments), and LysoTracker Green (for TatU1A-Alexa546-localization analyses) were combined in T buffer containing 0.1% polyvinyl alcohol (PVA; Sigma-Aldrich, St. Louis, MO, USA) and incubated at 37 °C for 10 min. Mouse embryos were treated at 37 °C for 5 min with pronase (Sigma-Aldrich) to remove the zona pellucida. After thoroughly washing nine times with T buffer containing 0.1% PVA, the zona-free embryos were dropped into droplets of the abovementioned TatU1A-Alexa546/shRNA solution (5 µL) in a dish covered with paraffin oil, and incubated at 37 °C under a 5% CO_2_ atmosphere for 3 h. Embryos were washed three times with Hepes-buffered KSOM (H-KSOM) medium and moved to a 35-mm glass-base dish filled with H-KSOM medium. A single blastomere of the embryo was irradiated via the photobleaching procedure using a laser at a wavelength of 543 nm on a confocal laser scanning microscope (FLUOVIEW FV-1000, Olympus, Japan). Photobleaching settings were as follows: type of bleaching: Clip Tornado, laser intensity: 20%, repetition: 200 frames. The embryos were then imaged using the confocal laser scanning microscope FLUOVIEW FV-1000.

### Preparation of mRNAs

Template DNAs for EGFP and BFP mRNAs were prepared via PCR using KOD Dash (TOYOBO, Japan) with 12XCSL-d1EGFP (Addgene, Watertown, MA, USA) and pTagBFP-N (Evrogen, Russia) vectors as templates. The template DNAs for EGFP and BFP mRNAs were amplified using the following primers (sequence of the T7 promoter is underlined): 5′-CCG GGT AAT ACG ACT CAC TAT AGG GAC ACA ACT GTG TTT ACT TGC-3′ and 5′-GAT GCT ATT GCT TTA TTT GTA AC-3′ for EGFP, and 5′-CCG GGT AAT ACG ACT CAC TAT AGG TCT ATA TAA GCA GAG CTG G-3′ and 5′-GTT AAC AAC AAC AAT TGC ATT C-3′ for BFP. Capped mRNAs were prepared via in vitro transcription using the mMESSAGE mMACHINE T7 Transcription Kit (Invitrogen) and a polyA addition with the Poly (A) Tailing Kit (Invitrogen) according to the manufacturer's protocols.

### Embryo microinjection

For EGFP knockdown experiments, EGFP and BFP mRNA was injected into blastomeres 1 h before the shGFP delivery by PCDR. The solution including 400 ng/µL each of EGFP and BFP mRNAs was loaded into glass micropipettes, and mRNA was injected into an arbitrary blastomere of 4-cell stage embryos using a piezo-driven micropipette unit (Prime Tech, Japan) and incubated at 37 °C for 1 h. Manipulation was carried out in M2 medium containing 5 mg/mL cytochalasin B (Sigma-Aldrich). The volumes injected typically ranged from 2 to 10 pL, which is 1–5% the volume of the cell.

## Results and discussion

### Endocytosis of TatU1A-Alexa546 in blastomeres

**Figure 1:**
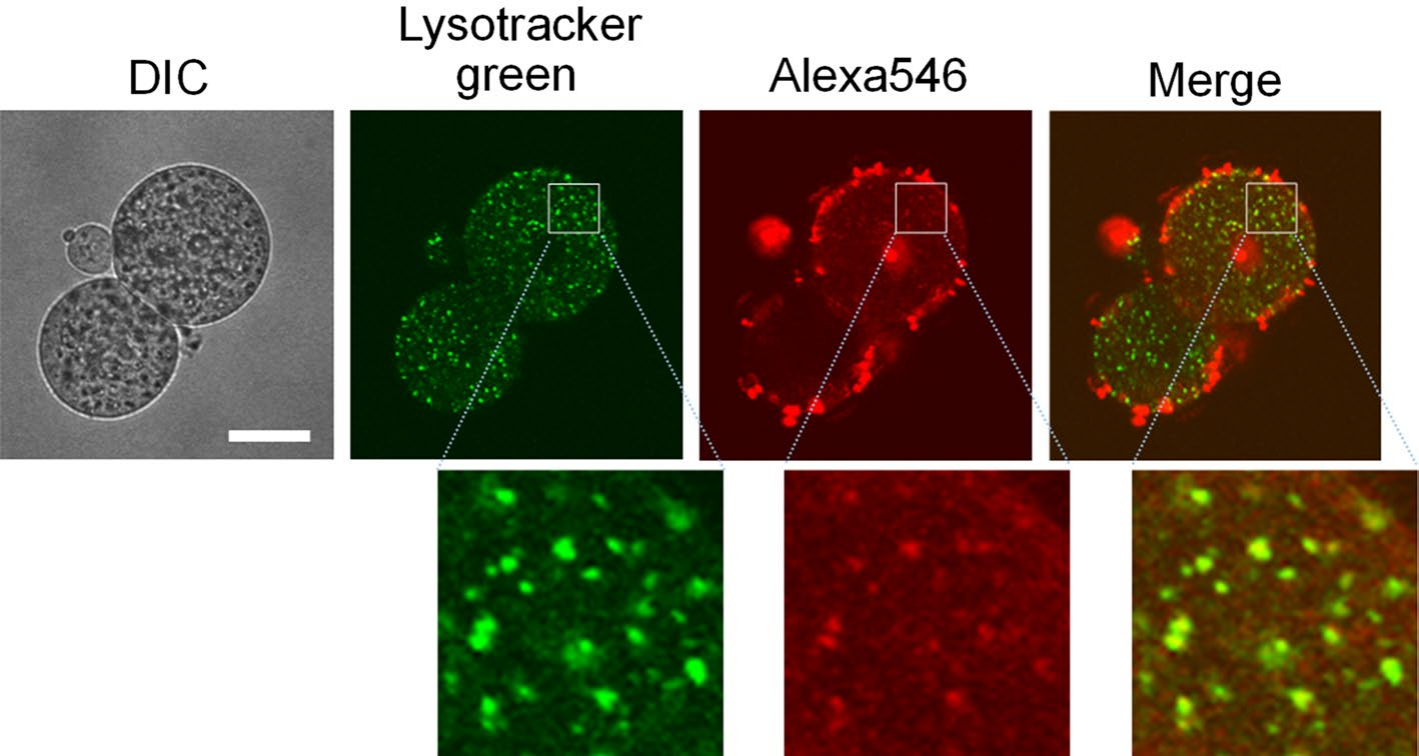
Representative image of co-localization of the TatU1A-Alexa546/RNA complex and LysoTracker green in a 2-cell stage embryo. We observed similar results in two more embryos. Scale bar, 30 μm.

The PCDR method requires the TatU1A-Alexa546/RNA complex to be endosomally entrapped before its photo-dependent cytosolic dispersion. Thus, blastomeres were stained with LysoTracker Green to confirm the subcellular localization of the transfected TatU1A-Alexa546/RNA complex. As a result, LysoTracker Green-stained acidic vesicles such as endosomes co-localized with Alexa546 (Fig. 1). This indicates the endo-lysosomal localization of TatU1A-Alexa546/RNA complex after the 3-h transfection procedure.

### Single blastomere-specific cytosolic RNA delivery

**Figure 2:**
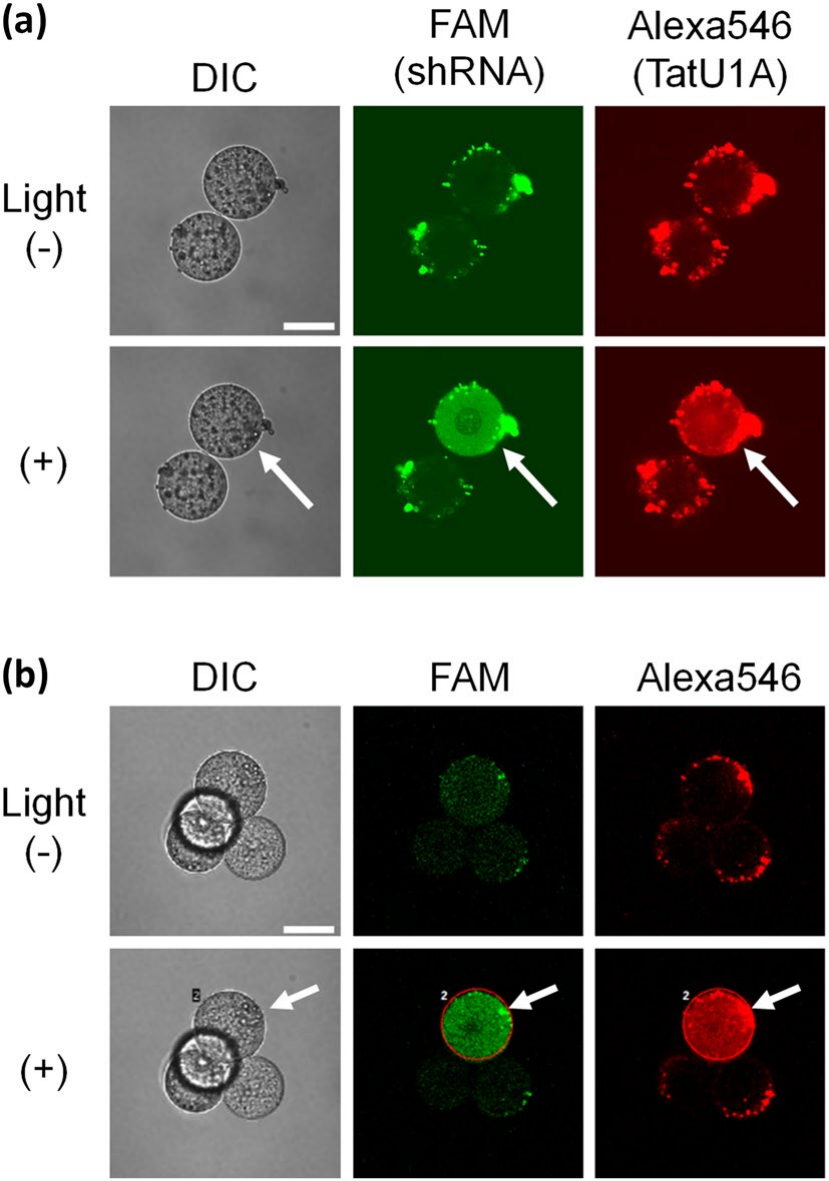
PCDR-mediated shRNA delivery into single blastomere in embryos. Irradiated blastomeres are indicated by arrows in the light (+) images. RNA delivery in 2-cell stage (**a**), and 4-cell stage (**b**) embryos are shown. Scale bar, 30 μm.

Mouse embryos were treated with the mixture of TatU1A-Alexa546 and shRNA after removing the zona pellucida, and a single blastomere was photoirradiated at the excitation wavelength of Alexa546. This procedure resulted in the dispersion of the FAM-labeled shRNA only within the irradiated blastomere (Fig. 2). The carrier, TatU1A-Alexa546, was also photo-dependently dispersed in the cytosol (Fig. 2a). The single blastomere-specific cytosolic shRNA delivery could be performed in 2-cell, 4-cell, and 8-cell stage embryos (Figs. 2a,b, and S1). Observations up to 2–4 days after light irradiation showed that the development of embryos had progressed to the morula stage (Figs. S1, S2). More than 70% (N = 32) of the irradiated embryos showed PCDR-mediated shRNA delivery and survived for more than two days (Table S1). Although we removed the zona pellucida before delivering the RNA, mouse zona-free embryos are generally known to develop without any problems through transfer and implantation. Zona pellucida is usually removed before viral transfection into fertilized eggs to create transgenic mice^20,21^. In addition, it has been reported that human embryos develop more effectively when the zona pellucida is removed^22^.

We also attempted a method of piercing two holes in the zona pellucida with a microinjection needle without removing it and allowing TatU1A-Alexa546 and shRNA to flow into the zona pellucida. Also in this case, shRNA delivery into photoirradiated single blastomere was observed (Fig. S2). The advantage of this method over the zona-free method is that it prevents the blastomeres from falling apart before compaction occurs. However, we mainly used zona-free embryos in this study because the two-holes method is more difficult and time-consuming than the zona-free method.

### Photo-mediated RNAi

**Figure 3:**
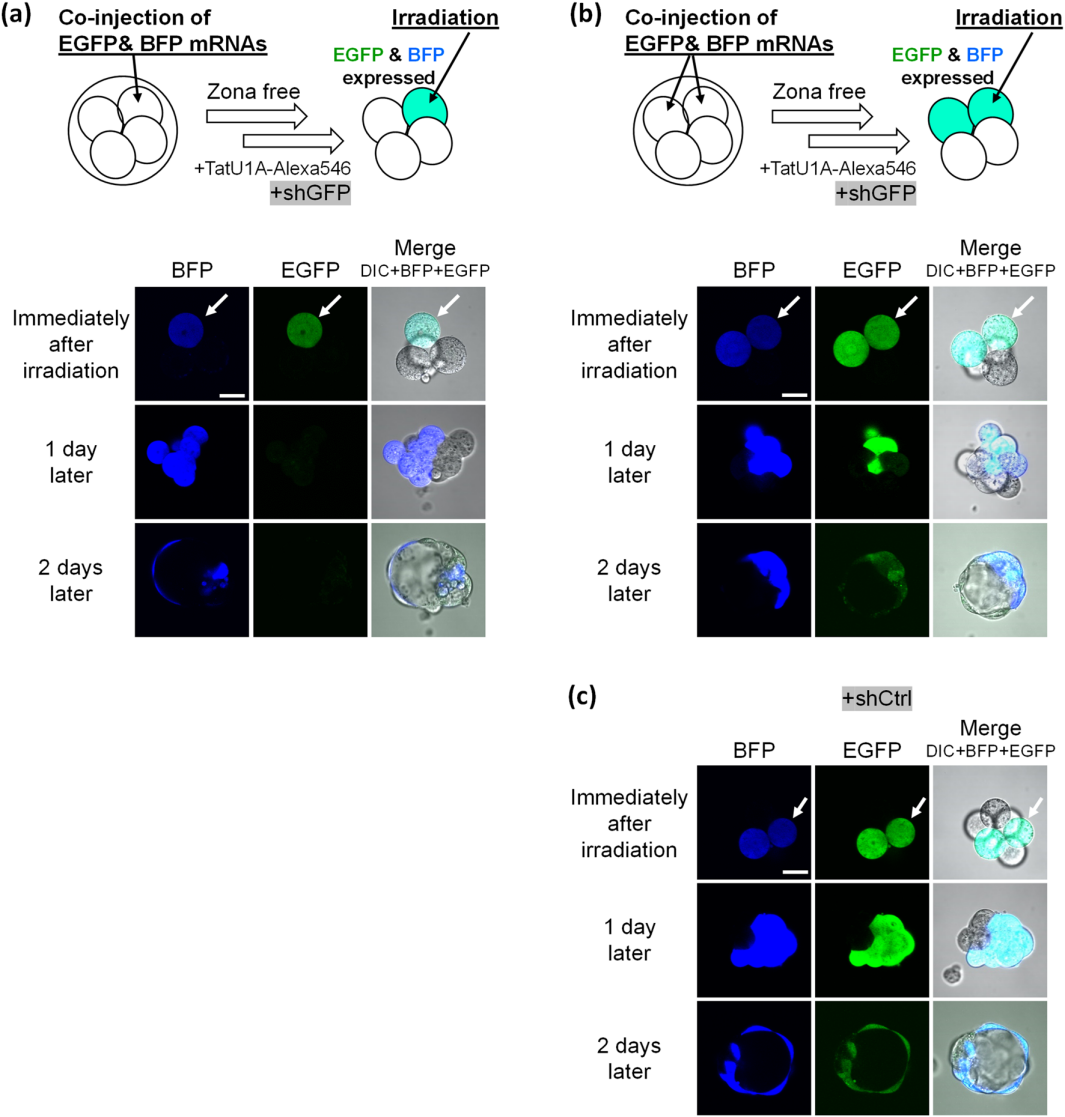
EGFP knockdown in a single blastomere. (**a**) One blastomere was co-injected with EGFP and BFP mRNAs, and photoirradiated to deliver shGFP. (**b**) Two blastomeres were co-injected with EGFP and BFP mRNAs, and one blastomere was photoirradiated to internalize shGFP. (**c**) Two blastomeres were co-injected with EGFP and BFP mRNAs, and one blastomere was photoirradiated to internalize shCtrl. We confirmed photo-dependent EGFP knockdown in six other embryos. Scale bar, 30 μm.

We then investigated RNAi with shRNA delivered by the PCDR method (Fig. 3). In 4-cell stage embryos, EGFP and BFP mRNA was microinjected into only one blastomere (Fig. 3a) or two blastomeres (Fig. 3b,c), and after removing the zona pellucida, the cells were incubated with TatU1A-Alexa546/shRNA for 3 h followed by photoirradiation at a single blastomere. EGFP knockdown was observed after photoirradiation when shGFP was used. When EGFP mRNA was injected into one cell prior to cell irradiation, the EGFP fluorescence completely disappeared after 1–2 days in 100% (N = 6) of the irradiated embryos (Fig. 3a, Table S2). This result indicates that EGFP knockdown was RNAi-mediated rather than via nonspecific translational repression because the injection marker BFP was still observable. The BFP used in this experiment (TagBFP derived from *Entacmaea quadricolor*) is not a homologue of EGFP derived from *Aequorea victoria*, and thus there is no concern of anti-EGFP shRNA misrecognizing BFP. When EGFP mRNA was injected into two cells and only one of the cells was irradiated, EGFP fluorescence disappeared in approximately half of the BFP fluorescent cells after 1 day (Fig. 3b). EGFP knockdown was not observed when shCtrl was used (Fig. 3c), indicating that the knockdown shown in Fig. 3a and b is dependent on shGFP.

In the experiment using control shRNA (Fig. 3c), we irradiated a single blastomere by the excitation light (543 nm) for the photosensitizer, and irradiated the entire embryo by excitation light for EGFP (488 nm) and BFP imaging (405 nm), but did not observe specific loss of EGFP. In addition, we observed that cells expressing both EGFP and BFP divided and increased in number after irradiation. This result indicates that these excitation lights neither induced EGFP-specific decrease nor cell death. We observed EGFP even 1–2 days after irradiation when shCtrl was used (Fig. 3c), which negates the possibility that difference in stability between EGFP and BFP caused the specific loss of EGFP in Fig. 3a,b. The expression sites of EGFP and BFP were perfectly identical after 1–2 days when shCtrl was used (Fig. 3c) which proved that the result seen in Fig. 3b (loss of EGFP in half of the BFP-expressing cells) could not have occurred because only BFP could diffuse into the adjacent cells.

In this study, the PCDR method was shown to work only with pre-injected synthetic mRNAs. In the case of chromosome-derived mRNAs, the duration of knockdown is likely to be shorter than for pre-injected mRNAs, even for the same mRNA, because the mRNA is produced again after shRNA introduction.

## Conclusion

This study demonstrates that the PCDR method can be used for shRNA delivery into a single blastomere in an embryo, and gene expression can be regulated by the delivered shRNA. According to our previous studies, the PCDR method is scalable in terms of usable light wavelengths and RNA species that can be delivered. In addition to the green light (543 nm) used here for photostimulation, red and near-infrared light can also be used in the PCDR strategy. We have previously demonstrated PCDR-mediated, light-dependent RNA delivery using several TatU1A-photosensitizer molecules with photosensitizers such as Alexa633 (λex ~ 633 nm; red) and DY750 (λex ~ 750 nm; near-infrared)^13,23^. The PCDR method enables the delivery of a precursor miRNA in addition to shRNAs and photoinduced apoptosis mediated by the miRNA^18^. The PCDR method is an efficient approach to the spatial regulation of gene expression^23^, and temporally limited RNA upregulation^19^. In the future, this method is expected to be applied to embryonic developmental research by controlling spatiotemporal gene/RNA functions. Clarifying the role of temporal and local gene expression in early embryonic development may help to elucidate the causes of infertility and the underlying principles for assisted reproductive technologies such as in vitro fertilization^24–26^.

### Supplementary Information

Supplementary Information.

## Data Availability

All data reported in this manuscript are available upon reasonable request by contacting the corresponding author.
