# Supplementary material for: Photo-dependent cytosolic delivery of shRNA into a single blastomere in a mouse embryo
Source: Sci Rep. 2023 Aug 11;13:13050. doi: 10.1038/s41598-023-40361-9 (PMC10421928; doi:10.1038/s41598-023-40361-9)
Supplement: Supplementary file 1 — Supplementary Information. [file 41598_2023_40361_MOESM1_ESM.pdf]

Supporting Information to:

Photo-dependent cytosolic delivery of RNA into a single blastomere in a mouse embryo

Yuka Ikawa[1], Takuya Wakai[2], Hiroaki Funahashi[2], Tet Htut Soe[1], Kazunori Watanabe[1], Takashi Ohtsuki[1,*]

[1] *Department of Interdisciplinary Science and Engineering in Health Systems, Okayama University, 3-1-1 Tsushimanaka, Okayama 700-8530, Japan.*

[2] *Department of Animal Science, Graduate of Environmental and Life Science, Okayama University, Okayama 700-8530, Japan.*

*To whom should be correspondence

Email: ohtsuk@okayama-u.ac.jp

Table S1. Number of embryos in the PCDR-mediated shRNA delivery experiments, which are represented by embryos shown in Figure 2 and S1

	Numbers of embryos
Irradiated	32
Embryos showing shRNA delivery	23
Embryos that survived for 2 days after irradiation	25

Table S2. Number of embryos in the EGFP knockdown experiments treated with shGFP or shCtrl, which are represented by embryos shown in Figure 3

	shGFP	shCtrl
mRNA-injected	6	3
Embryos that survived the injection	6	3
Irradiated	6	3
Embryos that survived for 2 days after irradiation	6	3
Embryos showing EGFP knockdown	6	0

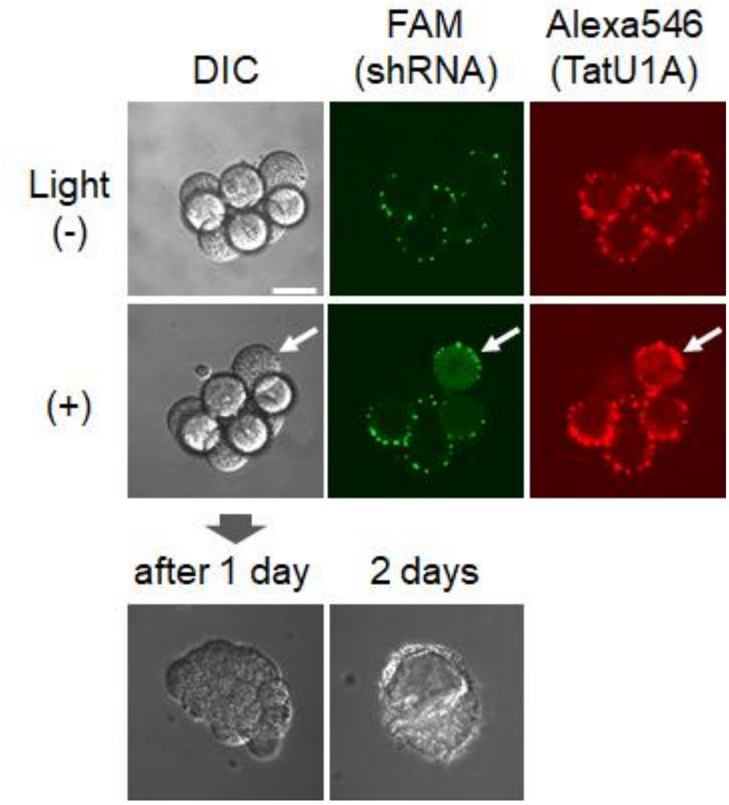

Supplementary Figure S1. Representative image of PCDR mediated shRNA delivery into a single blastomere in an 8-cell stage embryo. Irradiated blastomeres are indicated by arrows in the light (+) images. We observed similar results in four other embryos. Scale bar, 30 μm.

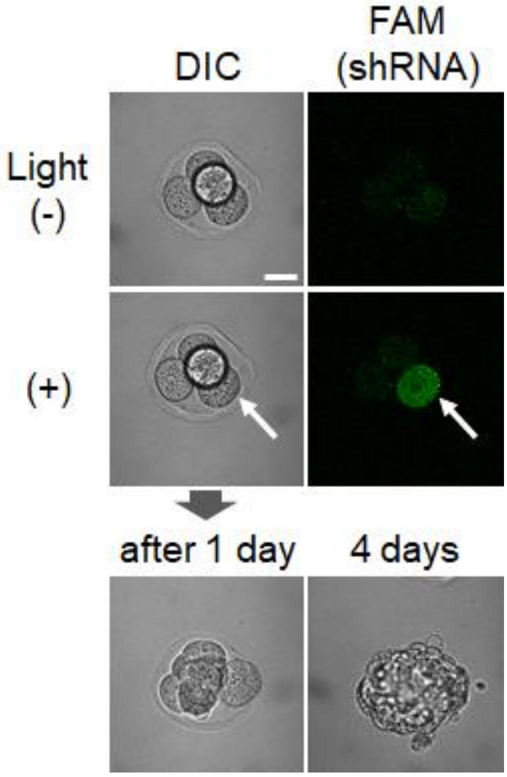

Supplementary Figure S2. Representative image of PCDR-mediated shRNA delivery into a single blastomere in a 4-cell stage embryo without removing the zona pellucida. Irradiated blastomeres are indicated by arrows in the light (+) images. We observed similar results in two other embryos. Scale bar, 30 µm.
